# Metabolites of *Moringa oleifera* Activate Physio-Biochemical Pathways for an Accelerated Functional Recovery after Sciatic Nerve Crush Injury in Mice

**DOI:** 10.3390/metabo12121242

**Published:** 2022-12-09

**Authors:** Muhammad Imran, Ghulam Hussain, Arruje Hameed, Iqra Iftikhar, Muhammad Ibrahim, Rahat Asghar, Izzat Nisar, Tahir Farooq, Tanzila Khalid, Kanwal Rehman, Mohammed A. Assiri

**Affiliations:** 1 Department of Chemistry, Faculty of Science, King Khalid University, Abha 61413, Saudi Arabia; 2 Reseach Center for Advanced Materials Science (RCAMS), King Khalid University, Abha 61514, Saudi Arabia; 3 Neurochemicalbiology and Genetics Laboratory (NGL), Department of Physiology, Faculty of Life Sciences, Government College University, Faisalabad 38000, Pakistan; 4 Department of Biochemistry, Faculty of life Sciences, Government College University, Faisalabad 38000, Pakistan; 5 Department of Applied Chemistry, Faculty of Physical Sciences, Government College University, Faisalabad 38000, Pakistan; 6 Department of Pharmacy, The Women University, Multan 60000, Pakistan

**Keywords:** sensory functions, motor functions, oxidative stress, peripheral nerve injury, nerve regeneration

## Abstract

In this study, the functional metabolites of *Moringa oleifera* (*MO*) were screened to evaluate their possible role in accelerated functional retrieval after peripheral nerve injury (PNI). *MO* leaves were used for extract preparation using solvents of different polarities. Each dry extract was uniformly mixed in rodents’ chow and supplemented daily at a dose rate of 2 g/kg body weight from the day of nerve crush until the completion of the trial. The sciatic functional index (SFI) and muscle grip strength were performed to assess the recovery of motor functions, whereas the hotplate test was performed to measure the regain of sensory functions. An optimal level of oxidative stress and a controlled glycemic level mediates a number of physio-biochemical pathways for the smooth progression of the regeneration process. Therefore, total oxidant status (TOS), total antioxidant capacity (TAC) and glycemic levels were analyzed in metabolite-enriched extract-treated groups compared to the control. The supplementation of polar extracts demonstrated a significantly high potential to induce the retrieval of sensory and motor functions. Further, they were highly effective in controlling oxidative stress, facilitating accelerated nerve generation. This study has highlighted *MO* as a sustainable source of nutritive metabolites and a valuable target for drug development.

## 1. Introduction

Plants are rich sources of nutritious phytochemicals that have always been under exploration to identify novel drugs against a range of ailments. Plant-based bioactive molecules have become promising candidates for modern drug development owing to their non-hazardous nature and the broad spectrum of therapeutic effects [1,2,3]. Phytochemicals such as curcumin, quercetin, capsaicin, resveratrol and taxol have shown antioxidant, antimicrobial, anti-inflammatory, anti-HCV and anticancer potential and thus are potent leads for alternative medicines. Innumerable plant-based natural products have shown their therapeutic potential against various brain and neurodegenerative diseases such as Alzheimer’s disease, Parkinson’s disease and Huntington’s disease [4,5,6].

PNI resulting from crushing, stretching and penetrating trauma or road accidents interrupts the normal sensory and motor functioning of nerves causing restricted movement to irreversible physical disability. PNI actually damages the integrity of Schwann cells and degenerates axons causing their functional loss. The injured nerve has the innate potential for regeneration but is usually a slow and complicated process and does not help in complete functional recovery [7,8]. A number of therapeutic agents are employed for an accelerated and complete regain of sensory and motor functions but generally they show limited success to support the regeneration process [9,10].

In recent years, efforts have been continuously made to explore new nutritional bioactive molecules potent enough to achieve full functional retrival after PNI; therefore, phytochemicals are of extreme interest due to their nonhazardous nature and safety profiles. The *MO* belonging to the family of Moringaceae is native to the Indian sub-continent and is commonly known as a ‘Miracle tree’ because of its nutritive values and medicinal importance. It is a source of various nutritious phytochemicals showing antimicrobial, antidiabetic, anti-inflammatory, anticancer and antioxidant properties [11,12,13]. *M. oleiferia* has emerged as an effective neuroprotectant due to its antioxidants. It helps to treat cerebral ischemia by controlling reactive oxygen species and regulating the flow of blood to the brain [14]. Its leaf extract could improve cholinergic function and memory by down regulating acetylcholine esterase activity used to treat dementia [15,16]. In a very recent study, the methanol extracts of *MO* regulated Ca levels to prevent mitochondrial dysfunctioning, upregulated antioxidants and level of glutathione to induce a neuroprotective effect in human neuroblastoma cells [17].

Considering these evidences, we envisioned a positive role of *MO* supplementation for accelerated nerve regeneration in the case of PNI. This experiential study was planned to evaluate the neuroregenerative capacity of *MO* and its effects on the regaining of motor and sensory functions following sciatic nerve crush injury in mice.

## 2. Material and Methods

### 2.1. Animals

Male albino mice weighing 28–32 g at an age of 9–10 weeks were obtained from the animal facility of the Department of Physiology, Government College University, Faisalabad. Under a standard control environment, they were kept in clean cages with one mouse per cage, and sub-divided as six treated groups (*n* = 6/group) and control (*n* = 6). Throughout this experiential study, they were exposed to 12 h of a dark/light cycle at 25 ± 2 °C and optimal humidity in the housing facility. All behavioral tests were performed in the light cycle at same time.

The study was carried out in accordance with the regulation and guidelines after the approval by the Institutional Review Board, Government College University, Faisalabad.

### 2.2. Extract Preparation and Supplementation

*M. oleifera* leaves were purchased from the local market of Faisalabad, Pakistan, and identified by the department of Botany, Government College University, Faisalabad. The leaves were shade dried, and grounded into fine powder before extract preparation using n-Hexane (n-Hex), dichloromethane (DCM), ethyl acetate (EtOAc), ethanol (EtOH) and methanol (MeOH) as solvents. Each dry extract was uniformly mixed in rodents’ chow at the daily dose rate of 2 g/kg body weight [18]. Each extract was supplemented to the separate group (treated group, *n* = 6) until the completion of trial. Food intake and average body mass were recorded everyday [19].

### 2.3. Induction of Sciatic Nerve Crush Injury

Well-established methods were adopted for sciatic nerve crush injury [20]. Under anesthesia, mice were induced with a sciatic nerve lesion surgically after the period of acclimatization. A mixture of Xylazine and Ketamine were injected intraperitoneally at the dose rate of 5 mg/kg and 70 mg/kg body weight, respectively. An incision was made in the skin (clearly shaved off) on the right thigh of the mouse to expose the thigh muscles. With a pair of forceps, the muscles were separated carefully and the sciatic nerve was exposed and compressed for 15 s. Next, the skin was sutured and disinfection. After the induction of sciatic nerve injury in all mice, they were divided into the following groups: normal chow group as control, the crude *MO* chow group, n-Hexane extract chow group, Dichloromethane extract chow group, ethyl acetate extract chow group, ethanol extract chow group and methanol extract chow group. Tissue samples were harvested along with the collection of blood samples after the decapitation of mice upon the completion of experiment.

### 2.4. Behavioral Tests

#### 2.4.1. Sciatic Functional Index

The recovery of motor function is measured by the SFI by employing a well-known procedure [21,22]. The mice with ink-painted hind paws were allowed to walk to a specified point on a wooden track. The SFI for control and treated groups was measured from paw prints using following formula,

$$\mathrm{SFI}=\left(-38.5\times \frac{\mathrm{EPL}-\mathrm{NPL}}{\mathrm{NPL}}\right)+\left(109.5\times \frac{\mathrm{ETS}-\mathrm{NTS}}{\mathrm{NTS}}\right)+\left(13.3\times \frac{\mathrm{EIT}-\mathrm{NIT}}{\mathrm{NIT}}\right)-8.8$$

Here EPL = Experimental print length, NPL = Normal print length, ETS = Experimental toe spread, NTS = normal toe spread, EIT = Experimental intermediate toe, NIT = normal intermediate

#### 2.4.2. Hot Plate Test

The hot plate test was employed to measure the hind limbs’ sensory function recovery of the mice. According to the procedure, mice were subjected to standing with their operated hind paw on the surface of a hot plate (55 ± 2 °C) until they displayed any response. This time was noted as the hot plate latency (HPL) period using a stopwatch, and 3 readings were recorded with a time gap of 2 min for every subject [23].

#### 2.4.3. Measurement of Grip Strength of Muscle

The ability of mice to clamp a horizontal grid or metal bar has been employed for the evaluation of muscle strength using a grip strength meter. Mice were placed on a metal grid and permitted to spontaneously grasp to stop the unintentional backward movements made by the investigator. The mice did so until the withdrawing strength weakened their grip. The strength meter marked the peak of the animal pulling force and three readings were recorded for the ipsilateral, contralateral and hind limbs relative to the lesion.

### 2.5. Biochemical Analysis of Systemic Indexes

#### 2.5.1. Total Antioxidant Capacity

The TAC measurement helps in the evaluation of the antioxidant potential of the biosystems. The well-established TAC assay works on the principle of the formation of a free radical of 2,2′-azinobis 3 ethylbenzothiazoline-6-sulfonate (ABTS) following its incubation with H_2_O_2_ [24]. The TAC measurements are expressed in mmol Trolox Equiv/L. The Trolox solution (5 mmol/L) was employed as a standard for the measurement of antioxidants in the serum samples using a semi-automated chemistry analyzer (Biosystem, BTS-330, Barcelona, Spain) [25].

#### 2.5.2. Total Oxidant Status

In this study, the TOS was measured by employing a well-known method based on the oxidation of Fe^2+^ into Fe^3+^ in the presence of an oxidant and an acid. The oxidation process was followed using xylenol orange with a semi-automated chemistry analyzer (Biosystem, BTS-330) to evaluate the oxidant status in the serum sample [26].

#### 2.5.3. Random Blood Glucose

The glycemic level in the control and extract chow groups was measured by using the glucometer (Accu-chek). In this test, a tiny drop of blood was taken from the tail of the mice [21]. The measurement of glycemic levels helps to interpret the biochemical pathways mediating the functional recovery and regeneration process.

## 3. Results

### 3.1. Effects of M. oleifera Extracts on Food Consumption and Body Mass

**Figure 1:**
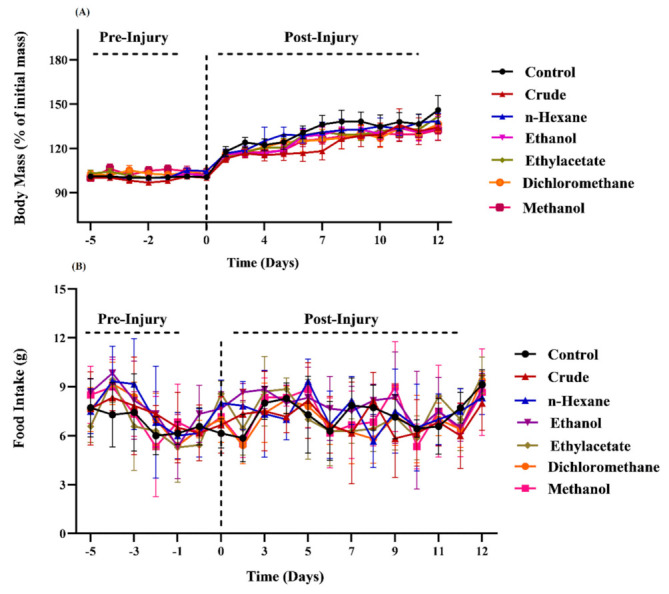
Impact of *M. oleifera* on (**A**) mouse’s body mass (**B**) food intake pattern.

The body mass and food intake were measured and compared throughout the study period (before and after inducing the sciatic nerve injury) in the control and extract chow groups as shown in Figure 1A,B. A statistically non-significant difference between groups on all days suggests that the addition of crude and extracts of *MO* and in the mice’s diets did not affect their eating behavior because granularity, smell and taste remained unchanged. Further, the supplementation did not cause a significant change in mice’ metabolism pattern because the body mass of animals increased relatively with non-significant differences between the control and treated groups.

### 3.2. Effects of M. oleifera on Muscle Strength and Retrieval of Motor Functions

**Figure 2:**
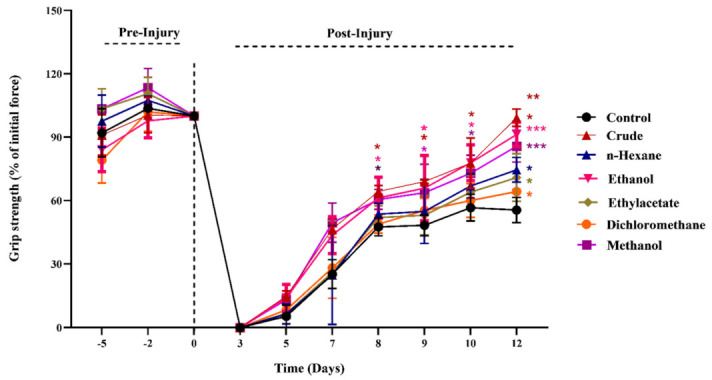
Influence of *M. oleifera* on muscle grip strength following sciatic nerve lesion. *, **, *** show the significance of results statistically.

The measurement of the SFI and muscle grip strength at different time points pre- and post-injury helps to evaluate the recovery of motor functions. The grip strength analysis revealed that *MO*-treated groups exhibited an accelerated retrieval of gripping force compared to the untreated control (Figure 2). However, a significantly high recovery of muscle strength was induced by the chow with crude, methanol and ethanol extracts from days 3 to 12 after injury, whereas the groups treated with extracts of relatively nonpolar solvents such as n-Hex, DCM and EtOAc caused a somewhat similar boosting effect on retrieval of muscle strength from days 9 to 12 compared to the control.

**Figure 3:**
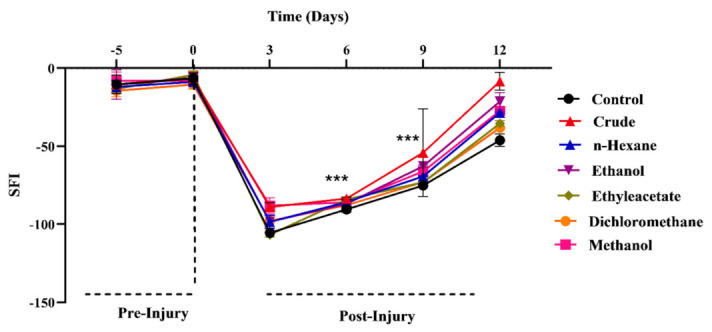
Influence of *M. oleifera* on SFI following sciatic nerve lesion. *** show the significance of results statistically.

The SFI values improved significantly from post-injury days 6 to 12 in treated groups compared to control (Figure 3). Again, the chow containing crude powder, ethanol and methanol extract caused a significant improvement in the SFI compared to the control group. However, in the case of nonpolar extracts, the n-Hex chow group displayed a relatively improved SFI than that of the DCM and EtOAc chow groups on day 12. Overall, all treated groups exhibited significant improvement in the SFI compared to the untreated ones. Thus, an improvement in the SFI corresponds to an accelerated recovery after nerve injury.

### 3.3. Effects of M. oleifera on Regain of Sensory Functions

**Figure 4:**
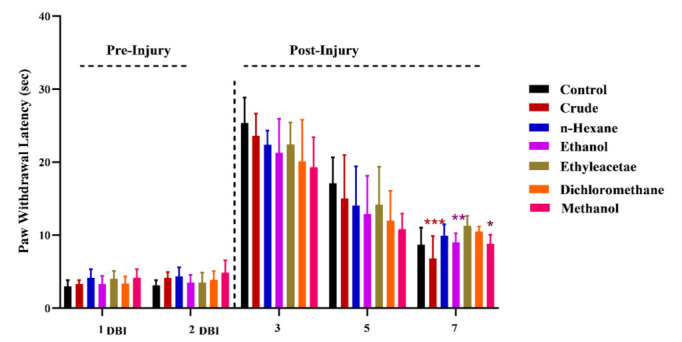
Effects of *M. oleifera* on recovery of sensory function following sciatic nerve lesion. *, **, *** show the significance of results statistically.

Sensory and motor functions are lost after sciatic nerve injury. In this study, formalin and hotplate tests were conducted at different time points both before and after nerve injury to assess the regaining of sensory functions in control and *MO*-treated mice. The results revealed a significant restoration of sensory response in *MO*-treated groups compared to control, as reflected by earlier paw withdrawal from the hot surface at day 7 post-injury (Figure 4). This indicated the positive effects of *MO* for an accelerated retrieval of sensory functions after nerve injury.

### 3.4. Effects of M. oleifera on Oxidative Stress and Blood Glucose Level

**Figure 5:**
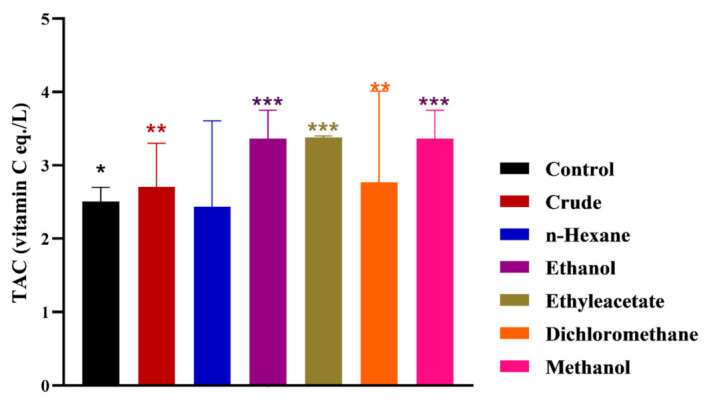
Effects of *M. oleifera* on total antioxidant capacity following sciatic nerve lesion. *, **, *** show the significance of results statistically.

**Figure 6:**
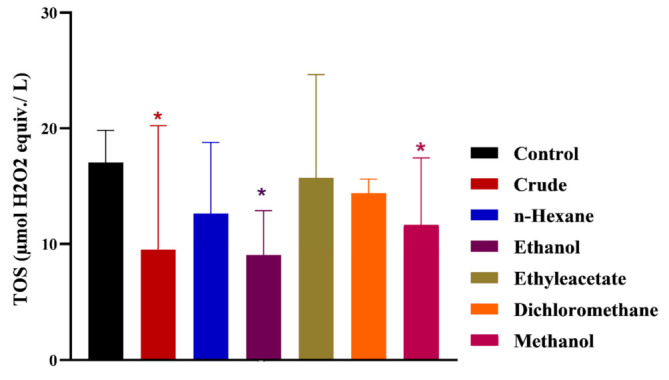
Effects of *M. oleifera* on total oxidant status following sciatic nerve lesion. * show the significance of results statistically.

After nerve injury, the development of local oxidative stress generally slows down the process of functional recovery. In this study, the TAC and TOS were measured in *MO*-treated and control groups for an assessment of the influence of phytochemicals on oxidative stress. All treated group exhibited significantly high TAC compared to the control (Figure 5). However, the ethyl acetate, ethanol and methanol chow groups showed maximum TAC whereas the n-Hex group induced minimum TAC, thus increasing impact. In TOS measurement studies, all treated groups displayed significantly low levels compared to control (Figure 6). However, among the treated groups, the crude, ethanol and methanol chow groups exhibited minimum TOS, whereas the n-Hex, DCM and ethyl acetate chow groups were at a relatively higher status.

### 3.5. Effects of M. oleifera on Glycemic Level following Sciatic Nerve Lesion

**Figure 7:**
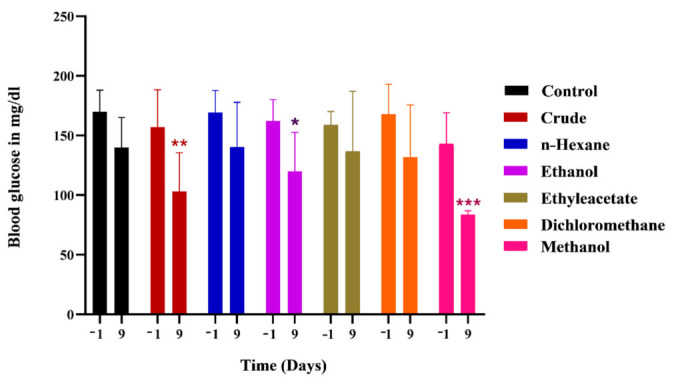
Effects of *M. oleferia* on glycemic levels (mg/dl) following sciatic nerve lesion. *, **, *** show the significance of results statistically.

All *MO*-treated groups showed significantly low glycemic levels compared to control on day 9 post injury (Figure 7), whereas, the minimum glycemic level was detected in the methanol chow group.

## 4. Discussion

Plant-based natural products have become attractive sustainable sources with immense potential for disease management beyond their traditional usage as nutrients. *M. oleifera* is enriched with a variety of structurally diverse phytochemicals and micro- and macronutrients, demonstrating a broad spectrum of therapeutic properties. In this study, different leaf extracts of *MO* were tested for their comparative efficacy to boost the recovery of sensory and motor functions after sciatic nerve injury. Different extracts were expected to provide structurally novel phytochemicals with unique neuroprotective and nerve regenerative capacities in peripheral nerve lesions.

The supplementation of crude leaves and dry extracts in mice chow did not affect their eating behavior. Furthermore, all treated groups experienced a significant increase in body mass compared to control, signifying the nutritional value of phytochemicals and their positive role in the modulation of metabolism under stress [27,28,29]. However, the maximum increase with a non-significant difference was induced by crude powder, ethyl acetate and methanol extracts on day 12 post-injury. The positive impact on body weight has been attributed to the phytochemical and nutrient reserves available in extracts mixed with chow. *M. oleferia* leaves carry seven times more vitamin C than oranges, more calcium than milk and higher contents of essential amino acids and proteins [30,31,32].

The grip strength analyses revealed the positive effects of *MO* on the recovery of motor functions after peripheral nerve lesion. The groups fed with crude, ethanol and methanol extracts exhibited a significantly high and maximum grip strength from days 5 to 12 post-injury compared to the control. The other three groups fed with relatively less-polar extracts exhibited an enhanced retrieval of motor functions from days 9 to 12 post-injury. This clearly indicated a different efficacy level and the potential of the phytoconstituents of *MO* for mediating the regaining of motor functions after sciatic nerve injury. The maximum retrieval of motor functions were induced by the supplementation of crude, possibly because of the synergistic effect of metabolites of various natures. In an earlier study, the dexamethasone-induced impairments in skeletal muscles were recovered with the accelerated retrieval of motor functions after supplementation with *MO* [33].

All treated groups exhibited a significantly high improvement in the SFI compared to the untreated ones. Again, the chow containing crude powder, ethanol and methanol extract caused maximum improvement of the SFI whereas nonpolar extracts induced relatively fewer improving effects. This again highlighted the polar phytoconstituents of *MO* as the key to regulate the recovery process of motor functions during the regeneration of nerves. Similarly, dietary supplementation of plant-based phenolics improved SFI scores, inducing an accelerated recovery after PNI [34,35,36].

Furthermore, the results of paw withdrawal latency time using hotplate tests revealed the regaining of sensory functions as a result of *MO* supplementation. A significant improvement in *MO*-mediated recovery of sensory functions was observed even on day 3 post-injury. The maximum recovery of sensory functions was observed in groups fed with chow containing crude, methanol or ethanol extract on day 7 post-injury. Needless to say, the observed sensory functions in control on day 3 pre-injury were same as in the ethanol extract-treated group on day 7 post-injury, which signified the crucial role of *MO*-based polar constituents in the retrieval of fully functional nerves after sciatic injury.

An optimal level of oxidative stress regulates a myriad of signaling pathways and physiological functions in nerve cells. Nerve injuries result in increased oxidative stress, which retards functional recovery and the regeneration process. Therefore, higher levels of antioxidant capacity and reduced oxidative status promote peripheral nerve regeneration after injury. TAC and TOS analysis revealed the level of oxidation stress and mechanistic insight into the recovery of damaged nerves. In our case, the TAC analyses revealed that all *MO*-treated groups exhibited a significantly higher level compared to the control. The groups supplemented with ethyl acetate, methanol and ethanol extract showed maximum TAC levels. The n-Hex extract was supposed to carry nonpolar constituents, generally showing low antioxidant potential. Therefore, supplementation of n-Hex extract did not produce any significant change in TAC compared to the control. Further, a significantly reduced TOS was detected in all *MO*-treated groups compared to the control. Thus, an increased TAC and reduced TOS in *MO*-treated groups ensures controlled oxidation stress, favoring the recovery process after nerve injury. Antioxidants facilitate the mechanism of the nerve recovery process by supplying metabolites and secondly by providing oxidative protection [37,38,39]. Furthermore, natural polyphenolic compounds have a well-established role in the functional recovery of peripheral nerve regeneration [36,40]. Various extracts of *MO* have well-studied antioxidant potential with a potent role in regulating a myriad of enzyme-controlled biochemical pathways [41,42,43]. A report described the reversal of fructose-induced insulin resistance in response to the application of MO extracts [44]. The phytoconstituents of *MO* controlled oxidative stress and protected liver mitochondria from the deleterious effects of oxidants [45]. *MO* leaf extract has shown the potential to control the antioxidant system in skeletal muscle cells regulating Nrf2-HO-1 pathways to avoid fatigue for the growth and repair of muscles [46,47]. Accordingly, in our case, MO leaf extracts are enriched with polyphenols and flavonoids, which control oxidative stress and expedite the functional retrieval of injured nerves.

The *MO*-treated group exhibited controlling effects on glycemic levels, which ultimately favors the regaining of sensory and motor functions and also the regeneration process after peripheral nerve injury. It has the capacity to control blood sugars and lowers glucose levels under normal and stress conditions [48]. The imbalanced glucose level impairs the nerve regeneration process by impeding a number of underlying biochemical mechanisms that support axon regrowth [49]. Various phytoconstituents including flavonoids and polyphenols are known to regulate glycemic level and oxidative stress, favoring the improvement of motor and sensory functions for the regeneration of peripheral nerves after injury [9,36,50]. *MO* leaves are enriched with polysaccharides, flavonoids and other polyphenolic compounds that enhance insulin sensitivity, control insulin levels and regulate metabolic pathways for a controlled blood glucose level [28,51].

The phytoconstituents in *MO*-leaf extracts induced an increase in body mass, improved muscle strength and enhanced sensory functions. All these positive impacts facilitated the regaining of sensory and motor functions after sciatic nerve injury. Furthermore, they controlled oxidative stress by increasing TAC and decreasing TOS and controlled glycemic levels for accelerated nerve regeneration.

## 5. Conclusions

*M. oleifera* extracts supplemented with chow accelerated the functional retrieval of the injured sciatic nerve. The extracts prepared using polar solvents exhibited a significantly high potential to induce the recovery of motor and sensory functions. They also showed a significantly high potential to control oxidative stress for accelerated nerve generation. The supplementation of crude dry powder leaves also exhibited a high potential to promote the regeneration process after peripheral nerve injury. This study has highlighted M. oleifera as a sustainable source of functional metabolites, and thus, as a valuable target for drug development in future clinical applications.

## Data Availability

The datasets used and analyzed during the current study are included in this manuscript.

## Abbreviations

PNI: Peripheral nerve injury; HPL: Hot plate latency; WRL: Withdrawal reflex; TAC: Total antioxidant capacity; TOS: Total oxidant status; TEAC: Trolox equivalent antioxidant capacity; ABTS: 2, 2′-azinobis 3 ethylbenzothiazoline-6-sulfonate.
